# Experimental and theoretical correlations between vanadium K-edge X-ray absorption and K$\varvec{\beta}$ emission spectra

**DOI:** 10.1007/s00775-016-1358-7

**Published:** 2016-06-01

**Authors:** Julian A. Rees, Aleksandra Wandzilak, Dimitrios Maganas, Nicole I. C. Wurster, Stefan Hugenbruch, Joanna K. Kowalska, Christopher J. Pollock, Frederico A. Lima, Kenneth D. Finkelstein, Serena DeBeer

**Affiliations:** 1 Max Planck Institute for Chemical Energy Conversion, Stiftstr. 34-36, 45470 Mülheim an der Ruhr, Germany; 2 Department of Chemistry, University of Washington, Box 351700, Seattle, WA 98195-1700 USA; 3 Faculty of Physics and Applied Computer Science, AGH University of Science and Technology, al. Mickiewicza 30, 30-059 Kraków, Poland; 4 Department of Chemistry and Chemical Biology, Cornell University, Ithaca, NY 14853 USA; 5 Centro Nacional de Pesquisa em Energia e Materiais, Laboratório Nacional de Luz Síncrotron, Rua Giuseppe Máximo Scolfaro 10000, Campinas, SP 13083-970 Brazil; 6 Cornell High Energy Synchrotron Source, Wilson Laboratory, Cornell University, Ithaca, NY 14853 USA; 7 Department of Chemistry, The Pennsylvania State University, University Park, PA 16802 USA

**Keywords:** Vanadium, X-ray spectroscopy, Density functional theory DFT, XES, XAS

## Abstract

A series of vanadium compounds was studied by K-edge X-ray absorption (XAS) and K$\beta$ X-ray emission spectroscopies (XES). Qualitative trends within the datasets, as well as comparisons between the XAS and XES data, illustrate the information content of both methods. The complementary nature of the chemical insight highlights the success of this dual-technique approach in characterizing both the structural and electronic properties of vanadium sites. In particular, and in contrast to XAS or extended X-ray absorption fine structure (EXAFS), we demonstrate that valence-to-core XES is capable of differentiating between ligating atoms with the same identity but different bonding character. Finally, density functional theory (DFT) and time-dependent DFT calculations enable a more detailed, quantitative interpretation of the data. We also establish correction factors for the computational protocols through calibration to experiment. These hard X-ray methods can probe vanadium ions in any oxidation or spin state, and can readily be applied to sample environments ranging from solid-phase catalysts to biological samples in frozen solution. Thus, the combined XAS and XES approach, coupled with DFT calculations, provides a robust tool for the study of vanadium atoms in bioinorganic chemistry.

**Electronic supplementary material:**

The online version of this article (doi:10.1007/s00775-016-1358-7) contains supplementary material, which is available to authorized users.

## Introduction

Vanadium plays an essential role in both biochemical and industrial catalysis. A number of solid-state catalysts employ vanadium in various oxide-type formulations, notably for the oxidative dehydrogenation of short chain alkanes; a highly desirable chemical transformation [1, 2]. While the natural abundance of vanadium is only 0.015 % in the earth’s crust, it is found in the oceans at concentrations as high as 30 nM, making it readily bioavailable [3]. At physiological conditions, vanadium is stable in the +3 to +5 oxidation states, and in its most oxidized forms is a potent Lewis acid. It is most commonly found in biology as some form of the vanadate ion, ${\mathrm{VO_{4}^{3-}}}$, though some enzymes that promote mostly oxidative transformations require vanadium as a metallocofactor [4–6].

For example, vanadium-dependent haloperoxidases promote the two-electron oxidation of halides using hydrogen peroxide as the terminal oxidant, or, in the absence of an organic substrate, can perform halide-dependent catalase-like disproportionation of hydrogen peroxide to water and dioxygen [7]. These haloperoxidases can also catalyze the oxidation of thioethers to sulfoxides, exhibiting oxo-transfer reactivity that is well established for high-valent vanadium complexes [8]. Interestingly, this sulfoxidation chemistry is the inverse of the reaction catalyzed by some molybdenum-dependent oxotransferases, such as DMSO reductase, which generate thioethers from the corresponding sulfoxide substrate at a molybdopterin active site [3].

The diagonal relation of molybdenum and vanadium and their use in similar, albeit complementary, biochemical sulfoxidation reactivity serves to further highlight their involvement in the promotion of arguably nature’s most complex chemical transformation: the six-electron reduction of aerial dinitrogen. Biological nitrogen fixation is catalyzed by the nitrogenase enzymes, which contain three distinct types of [Fe–S] clusters. A [4Fe–4S] cluster in the Fe protein provides reducing equivalents via hydrolysis of ATP, while in the MoFe or VFe protein an [8Fe–7S] P-cluster serves as an electron transfer relay, reducing the active site [M–7Fe–9S–C] cofactor, where M=Mo or V [9–14]. Extensive studies of these enzymes using a range of spectroscopic techniques continue to provide novel insight into the structure and function of nitrogenase; however, significant questions such as the role of the heterometal or the location of ${\mathrm{N_2}}$ binding still remain unanswered [12, 15–19].

In many of these enzymes, the identities of important intermediates, often including the catalytically competent species, are still unknown. Characterization of the spin state, oxidation state, and coordination environment of vanadium in a catalytic cycle are important prerequisites for mechanistic understanding. Existing methods often utilized in such studies include ${\mathrm{^{51}V}}$ nuclear magnetic resonance (NMR) [20] and various pulsed electron paramagnetic resonance (EPR) spectroscopies, [21, 22] as well as X-ray-based methods: vanadium K- and L-edge X-ray absorption spectroscopy (XAS) and extended X-ray absorption fine structure (EXAFS) [23–25]. Some of these techniques have limited applicability to enzyme systems, however. For example, L-edge XAS experiments are conducted in high-vacuum environments not ordinarily amenable to biological samples. Furthermore, as magnetic resonance methods have specific spin-state requirements, certain intermediates may be spectroscopically “silent”, rendering NMR and EPR unsuitable to probe certain steps during a catalytic cycle. Thus, the advancement of additional biologically amenable spectroscopic techniques, particularly those capable of probing a variety of changes in electronic structure without preference for oxidation or spin state, is highly desirable. Herein, we describe a combined X-ray spectroscopic study, utilizing both V K-edge XAS and K$\beta$ X-ray emission spectroscopies (XES). Calibration to density functional theory (DFT) calculations provides an insightful, element-specific probe of vanadium geometric and electronic structure, using experimental methods well adapted to the study of biological molecules.

In K-edge XAS, a 1*s* electron is excited into either bound states or to the continuum. In the former case, transitions into singly or unoccupied 3*d* orbitals of a first-row transition metal give rise to the pre-edge region, which can provide insight into the energies of the 3*d* manifold. Additionally, the intensities of these 1*s*$\rightarrow$ 3*d* transitions, while formally quadrupolar, are largely governed by metal *p*–*d* mixing, and thus the dipole selection rule [26, 27]. Changes in metal–ligand bond covalency [28] and geometric distortion from centrosymmetry both serve to modulate the intensity of the pre-edge transitions through perturbations in *p*–*d* mixing [29]. Time-dependent DFT (TD-DFT) calculations have proven capable of correctly predicting relative energies and intensities of these pre-edge features, and through careful analysis and comparison to experiment, additional insight into geometric and electronic structure can be obtained [26, 30, 31]. Even with the aid of theoretical insight, however, probing the electronic structure of ligand-based orbitals is a challenge for XAS, and in this area, XES holds significant promise.

Following the 1*s* ionization described above, in a one-electron picture, K$\beta$ emission is the fluorescent decay of an electron from the $n=3$ shell into a 1s core hole. In the case of first-row transition metals, for which these are the valence levels, the different regions in the K$\beta$ spectrum provide complementary chemical insight. The K$\beta$ mainline feature arises from metal 3*p*$\rightarrow$ 1*s* transitions, and is sensitive to the oxidation state and local spin at the absorber atom [32–34]. This dipole-allowed transition is intense, and is readily observable in low concentrations of metal atom emitter. The K-beta mainline has seen extensive prior use as a marker for oxidation and spin state, as the number of unpaired 3*d* electrons modulates the 3*p*–3*d* exchange coupling, and thus the multiplet-derived energetic splitting of the K$\beta$ mainline peaks. However, care must be taken in the interpretation of this splitting, as we have recently shown that the spectral features can be significantly modulated by metal–ligand covalency [34–37].

To higher energy, the weaker valence-to-core (VtC) region of the K$\beta$ XES spectrum probes the valence shell, and corresponds to transitions from molecular orbitals (MOs) which are typically dominated by ligand character. Accordingly, the VtC region has been shown to have a high degree of sensitivity to the identity, nature, and electronic structure of the first coordination sphere of ligands [35, 38–46]. As expected from a simple MO picture, the highest energy K$\beta _{2,5}$ peaks are found to originate from ligand *p*-type orbitals, while the lower energy K$\beta ''$ features are ligand *s*-type in nature. It is important to note that the “donor” orbitals in these VtC transitions are in fact MOs, rather than atomic orbitals, and thus the transition energies reflect the energetic ordering found in an MO diagram [39, 47]. Similar to XAS transitions, the intensities of VtC spectra derive almost entirely from electric dipole transitions, and are dependent on the degree of metal *p* character mixed into the ligand MO [39]. Generally, those MOs which are more diffuse have more metal *p* character and intense spectral features, while more contracted MOs (typically the K$\beta ''$) have weaker transitions. This dependence on metal *p* mixing also makes the VtC features highly sensitive to changes in metal–ligand bond lengths.

The sensitivity of VtC XES to changes in ligand electronic structure is in stark contrast to the insight obtained from EXAFS. In the latter method, subtle perturbations to a given scatterer, such as substrate bond activation or protonation at a basic ligand site, must be inferred solely from changes in bond length. In addition, for scatterers with similar *Z*, resolving their identities or individual bond lengths can be challenging. However, VtC XES has demonstrably detected evidence of substrate activation, e.g., the cleavage of an ${\mathrm{N_2}}$ bond [48] and changes in NO speciation [49, 50] in homogeneous and heterogeneous iron systems. Additionally, we have used VtC XES in concert with XAS to study the activation of manganese peroxo and oxo adducts, and shown these methods to be capable of detecting both O–O bond activation [51] and the protonation of bridging oxo ligands [52, 53].

The present study couples standard vanadium K-edge XAS methods with TD-DFT calculations, and also explores the highly complementary chemical insight that can be derived from K$\beta$ XES and ground-state DFT calculations. A series of molecular and extended lattice compounds were studied (Table 1), and correlations have been made between XAS and XES spectral features, as well as experimental and calculated parameters.

**Table 1:** Vanadium compounds examined in this study, and relevant properties thereof

Compound	Oxidation state	*d* count	Coordination	Nominal symmetry	Ligand type(s)	Bond length^a^ (Å)
${\mathrm{Na_3VO_4}}$	V	0	Lattice (4)	${\mathrm{T_d}}$	V–O	1.71 (1.69–1.73)
${\mathrm{NaVO_3}}$	V	0	Lattice (4)	${\mathrm{T_d}}$	V–O	1.72 (1.64–1.81)
${\mathrm{V_2O_5}}$	V	0	Lattice (6)	${\mathrm{O_h}}$	V–O	1.96 (1.59–2.79)
${\mathrm{V_2O_4}}$	IV	1	Lattice (6)	${\mathrm{O_h}}$	V–O	1.93 (1.76–2.05)
${\mathrm{VO(acac)_2}}$	IV	1	5	${\mathrm{C4_v}}$	V–O, V=O	1.59, 1.99
${\mathrm{VCp_2Cl_2}}$	IV	1	4	dist. ${\mathrm{T_d}}$	V–Cp^b^, V–Cl	2.31, 2.41
${\mathrm{V_2O_3}}$	III	2	Lattice (6)	${\mathrm{O_h}}$	V–O	2.01 (1.96–2.07)
${\mathrm{VCl_3 * 3THF}}$	III	2	6	${\mathrm{O_h}}$	V–O, V–Cl	2.32, 2.08
${\mathrm{V(acac)_3}}$	III	2	6	${\mathrm{O_h}}$	V–O	1.98
${\mathrm{VCl_3}}$	III	2	Lattice (6)	${\mathrm{O_h}}$	V–Cl	2.42
${\mathrm{VCl_2}}$	II	3	Lattice (6)	${\mathrm{O_h}}$	V–Cl	2.53

^a^Entries are given as mean (range)

^b^ *Cp* $$\eta ^5$$-cyclopentadienyl

As combined XAS and XES studies have previously been utilized to understand simultaneous changes in both metal and ligand electronic structure in the case of iron [49, 54] and manganese [51–53], this study paves the way for similar investigations involving vanadium. We envision this XAS/XES approach as an ideal tool for the mechanistic investigation of vanadium-catalyzed reactions in both chemistry and biology, including the synthesis of halogenated biomolecules and the role of vanadium in the reduction of dinitrogen, promoted by vanadium nitrogenase.

## Experimental

All compounds are commercially available and were obtained from Aldrich except ${\mathrm{Na_3VO_4}}$, which was purchased from Spectrum. They were used without further purification, and when necessary samples were prepared, transported, and stored in an inert ${\mathrm{N_2}}$ atmosphere. Samples of all compounds were prepared by grinding the solid material into a fine powder in a mortar and pestle, which was then pressed into an aluminum spacer and sealed with 38 ${\upmu }$m Kapton tape. To minimize self-absorption during XAS measurements, samples were diluted approximately 9:1 by mass with boron nitride.

### Data collection

#### XAS

Vanadium K-edge XAS measurements were performed at the XAFS2 beamline at the Laboratório Nacional de Luz Síncrotron (LNLS) in Brazil, with a ring energy of 1.37 GeV and a current of 250 mA. Incident energy was selected using a Si(111) double-crystal monochromator, and focused using a Rh-coated cylindrical mirror to a beam spot of about $\mathrm{0.4 \times 0.4}$ mm^2^. Photon flux at the sample was approximately $\mathrm{10^{10}}$ photons/s. Samples were held below 80 K during measurements in a closed-cycle He cryostat. Spectra were collected in both transmission and fluorescence modes, with the former using a He/${\mathrm{N_{2}}}$-filled ion chambers and the latter utilizing an energy-resolving 15-element Ge detector (Canberra, Inc.). The fluorescence signal was obtained by integrating counts within a 170 eV window, centered at the V K$\alpha$ emission line (approximately 4.9 keV). Rapid scans over the XANES (X-ray Absorption Near Edge Structure) region were performed to screen for radiation damage, and data were collected and averaged from multiple sample spots so as to maintain a radiation dose per spot well below any observed damage threshold.

#### XES

Vanadium K$\beta$ XES measurements were performed at beamline C-1 at the Cornell High-Energy Synchrotron Source, with a ring current of 110 mA and ring energy of 5.3 GeV, operating in 90 min decay mode. The incident energy was set to approximately 9 keV using a pair of $\mathrm{W/{B_4C}}$ multilayers for a 1 % bandwidth. The beam spot size on the sample was about 2.0 $\times$ 1.0 mm$^2$, with an approximate flux of 2.6 $\times$ 10$^{12}$ photons/s on the sample, and during measurements, samples were maintained at approximately 40 K using a closed-cycle He cryostat. V K$\beta$ fluorescence was analyzed and collected using an array of three spherically bent Ge(422) crystal analyzers and a detector configured in a Rowland geometry. Data were initially collected using a Vortex Si drift detector (SDD); however, data analysis indicated that during measurements, the angular scanning motion of the crystal array caused a systematic shift in the focal points of the outer analyzer crystals, which resulted in artificially suppressed spectral intensity in the higher energy region. To correct for this suppression of intensity, a second set of experiments were performed using a spatially resolved Si pixel detector (Pilatus) in place of the SDD. This allowed for the visual monitoring of the transverse focal point of each crystal in the array, and optimization of the optical alignment throughout the entire range of spectrometer angles. Additionally, a digital region of interest (ROI) was chosen to tightly enclose the focal point, maximizing the signal to noise ratio (S/N). Finally, a larger, concentric ROI was chosen of four times the area, to collect and subsequently correct for background radiation.

### Data analysis

#### XAS

For each compound, between 4 and 11 transmission spectra were averaged to improve S/N. The pre- and post-edge background subtraction was performed using a second-order polynomial function, and the edge jump was normalized to unity. Experimental edge energies were obtained from plots of the second spectral moments. The pre-edge features were modeled with pseudo-Voigt peaks and fit with a least-squares regression, using BlueprintXAS [55], and pre-edge energies were determined from the intensity-weighted average energies (IWAEs) of the pre-edge peaks [26, 52]. Individual contributions of the Gaussian and Lorentzian functions to the pseudo-Voigt peaks did not exceed 80 %. The FWHM of all peaks were between 0.55 and 1 eV to represent the observed intrinsic transition linewidths. The integrated areas of the pre-edge peaks (Figures S1–S5) were summed to obtain the experimental pre-edge intensities reported in Table 2. An estimated error of 5 % is associated with the reported intensities due to pre- and post-edge background subtraction, normalization, and fitting [26, 30].

#### XES

Multiple scans of X-ray emission data were merged using PyMCA [56]. MATLAB was used for subsequent processing. The data were calibrated to the K$\beta _{1,3}$, K$\beta ''$, and K$\beta _{2,5}$ energies of ${\mathrm{V_2O_5}}$ reported by Jones and Urch (5426.3, 5448.1, and 5462.9 eV, respectively) [57]. The background was removed using the concentric ROI data, and the spectra were normalized to an integrated area of 1000. The experimental energies of the mainline and VtC features were determined from the first spectral moment. Each spectrum was then modeled using a sum of pseudo-Voigt profiles and least-squares fitting was performed to optimize the peak positions, intensities, FWHM, and percent composition of Gaussian and Lorentzian functions for each component profile. The peaks fitting the lower energy mainline region were subtracted to obtain a background-subtracted VtC spectrum. The integrated areas of the peaks in the VtC region (Figures S6–S9) were summed to obtain the experimental VtC intensities in Table 3. An estimated error of 10 % is associated with the reported intensities due to subtraction of the tailing K$\beta$ mainline, normalization, and fitting procedures [38, 40].

### Calculations

DFT and TD-DFT calculations were performed with the ORCA program package, v.3.0.3. [58]. Protocols similar to those we have previously established for the calculation of pre-edge XAS [26, 31] and VtC XES [38, 40] spectra were utilized. The present work uses the def2-TZVP basis and def2-TZV/J auxiliary basis sets of Ahlrichs and coworkers [59, 60]. Geometry optimizations, initiated from crystallographic coordinates available from the Cambridge Structural Database or the Inorganic Crystal Structure Database, as well as VtC XES calculations employed the BP86 functional [61, 62], and TD-DFT calculations of the XAS spectra used the B3LYP [62, 63] functional and the Tamm-Dancoff approximation [64, 65]. For all spectral calculations, a large integration grid was used on vanadium (Grid7), and tight SCF convergence criteria were required. For compounds possessing an extended lattice morphology, rather than molecular structures, a quantum cluster was selected for the DFT and TD-DFT calculations, and was embedded in a point charge field with a boundary region, as we have previously reported [66]. Calculated XAS and XES spectra were obtained by a 1 and 2 eV Gaussian broadening of the calculated combined transition moments, respectively, including electric and magnetic dipole and electric quadrupole contributions. Calculated spectral intensities and energies were determined from the sum of all combined transition moments in the spectral region and from IWAEs, respectively, as previously reported [26, 40, 52, 53]. Example input files are provided in the Supplemental Information.

## Results and discussion

This study aims to elucidate the complementary nature of the XAS and XES spectra herein, and to improve understanding of which methods and spectral regions offer what chemical insight. Additionally, correlations between fundamental chemical properties observed from both XAS and XES, as well as between experiment and theory, maximize the available information, and enhance the applicability of this work to future studies involving more complex chemical systems. To achieve these goals, two subsets of data have been chosen and discussed in detail below.

### XAS spectra

**Fig. 1:**
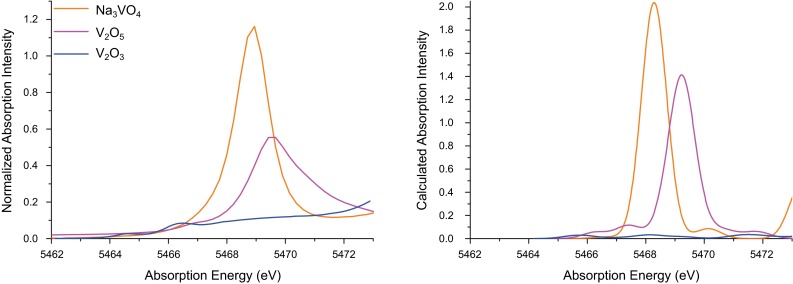
Experimental (*left*) and calculated (*right*) pre-edge regions of the XAS spectra of ${\mathrm{Na_3VO_4}}$, ${\mathrm{V_2O_5}}$, and ${\mathrm{V_2O_3}}$. Calculated pre-edge spectra are shifted by 115.1 eV

The pre-edge features in the XAS spectrum give the energies of the lower lying core-hole excited states. These correspond to 1*s*$\rightarrow$ 3*d* excitations in the case of first-row transition metals, and thus the pre-edge region can probe the energies and compositions of the unoccupied 3*d* orbitals. Figure 1 shows the pre-edge regions of the XAS spectra of ${\mathrm{Na_3VO_4}}$, ${\mathrm{V_2O_5}}$, and ${\mathrm{V_2O_3}}$, and marked differences in pre-edge energy and intensity are clearly visible. As reported in Table 2, a clear shift to higher edge energies for more oxidized compounds is also found, indicative of a larger vanadium 1s binding energy (full pre-edge and edge spectra are shown in Figures S1–S4) [25].

**Table 2:** Comparison of experimental and calculated XAS parameters

Compound	Experimental	Experimental pre-edge		Calculated pre-edge	
	Edge energy (eV)	Energy (eV)	Intensity	Energy (eV)	Intensity
${\mathrm{Na_3VO_4}}$	5481.8	5469.1	261	5469.3	262
${\mathrm{NaVO_3}}$	5482.0	5469.3	301	5469.6	300
${\mathrm{V_2O_5}}$	5480.5	5470.0	198	5470.1	227
${\mathrm{V_2O_4}}$	5477.1	5468.7	102	5470.0	41
${\mathrm{VO(acac)_2}}$	5480.8	5468.4	149	5468.7	198
${\mathrm{VCp_2Cl_2}}$	5475.7	5468.4	28	5467.5	4.0
${\mathrm{V_2O_3}}$	5475.0	5469.1	73	5469.8	18
${\mathrm{VCl_3 * 3THF}}$	5475.5	5467.4	26	5467.1	3.8
${\mathrm{V(acac)_3}}$	5479.3	5467.5	20	5467.3	7.2
${\mathrm{VCl_3}}$	5473.7	5467.5	31	5466.4	0.8
${\mathrm{VCl_2}}$	5471.2	5466.2	16	5466.0	1.3

Full edge regions are shown in the Supplemental Information, Figures S1–S5

Calculated IWAEs have been shifted by 115.1 eV and intensities are scaled by a factor of 9.7 from calibrations in Fig. 7

Total experimental intensities have been multiplied by 100

There is also a substantial range of total pre-edge intensities, or integrated spectral areas, determined by least-squares fitting (*vide supra*). As the intensity of the pre-edge is dominantly governed by the dipole selection rule, increased intensity corresponds to increased transition dipole moments [29, 67]. This can be understood in terms of increased mixing of vanadium *p*-orbital character into the 3*d* acceptor orbitals, and these data illustrate two key ways of modulating this *p*–*d* mixing.

In addition to changes in electronic structure, oxidation perturbs the structure of the oxide lattice. The vanadium ions occupy octahedral holes in the lattice, and upon oxidation the V–O bonds contract due to an increased Coulombic attraction. The shorter bonds promote *p*–*d* mixing, and thus a larger pre-edge intensity is observed for ${\mathrm{V_2O_5}}$ relative to ${\mathrm{V_2O_3}}$ [28, 68]. In contrast, ${\mathrm{V_2O_5}}$ and ${\mathrm{Na_3VO_4}}$ are both in the vanadium (V) oxidation state. While they have similar edge energies (Table 2), the pre-edge intensity of ${\mathrm{Na_3VO_4}}$ is substantially increased. The sodium cations intercalated in the ${\mathrm{Na_3VO_4}}$ lattice cause the vanadium ions to occupy tetrahedral sites, reducing the centrosymmetry at the vanadium center. The resultant increase in *p*–*d* mixing, here a function of geometry, increases the transition dipole moment and thus the pre-edge area.

**Fig. 2:**
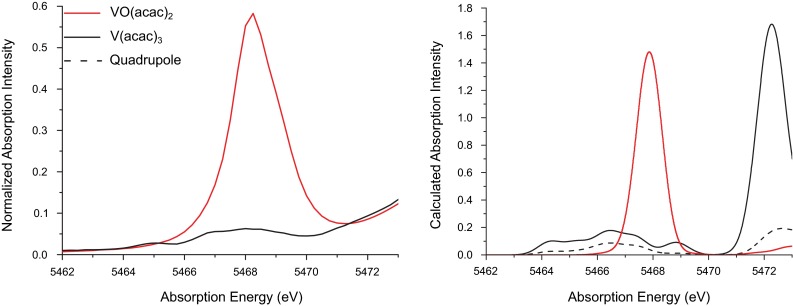
Experimental (*left*) and calculated (*right*) pre-edge regions of the XAS spectra of ${\mathrm{VO(acac)_2}}$ and ${\mathrm{V(acac)_3}}$. Calculated pre-edge spectra are shifted by 115.1 eV, the calculated ${\mathrm{V(acac)_3}}$ spectrum has been scaled up by a factor of 10 for clarity, and the quadrupole contribution to the pre-edge is shown as the *dashed trace*

The two molecular acetylacetonate (acac) complexes also present an interesting case study, as their related structures give rise to vastly different pre-edge spectra (Fig. 2).

${\mathrm{V(acac)_3}}$ is an octahedral complex ligated exclusively by carboxylate oxygens, and the small pre-edge intensity is typical of highly centrosymmetric metal centers. Without a mechanism for *p*–*d* mixing, the transition dipole moment is essentially zero. Accordingly, the calculated pre-edge transitions for the ${\mathrm{V(acac)_3}}$ spectrum shown in Fig. 2 are largely quadrupolar in origin (*vide infra*). However, the ${\mathrm{VO(acac)_2}}$ pre-edge has considerable dipolar intensity, which reflects substantial *p*–*d* mixing. Lacking a trigonal or tetrahedral ligand field, in this case the terminal oxo ligand induces *p*–*d* mixing due to the highly covalent bond it forms with vanadium, as well as a reduction in symmetry to ${\mathrm{C_{4v}}}$ [28, 68]. Of particular note is the inequivalence of the oxygen ligands’ influence on both the pre-edge spectrum and the underlying electronic structure; in short, there are two different “types” of oxygen ligands (the conjugated ${\mathrm{acac^{-}}}$ and the vanadyl ${\mathrm{O^{2-}}}$). Distinctions of this sort can, for example, be lost solely from interpretation of the EXAFS region, where only the ligand distance and approximate identity can be deduced. The pre-edge region clearly holds additional insight with regard to bonding and electronic structure, which can be understood using fundamental principles of coordination chemistry.

These data demonstrate the large range of pre-edge intensities possible for vanadium sites with similar oxidation states and coordination geometries. For example, the range of pre-edge intensities of octahedral vanadium ions spans over a factor of 20 from V(III) to V(V) ions (Table 2). Additionally, ${\mathrm{V(acac)_3}}$ and ${\mathrm{V_2O_3}}$ are both octahedral V(III) ions ligated exclusively by oxygen atoms, yet the pre-edge intensity of the latter is over three times greater. Changes in bond length, coordination geometry, and covalency are thus all found to have a profound influence on the intensity of the pre-edge features. As all of these factors can effectively modulate the extent of *p*–*d* mixing, it is impossible to isolate changes in pre-edge area to a given coordination number, metal geometry, or bond length. While relative changes within a series of structurally similar vanadium sites can be qualitatively insightful, more quantitative understanding relies on computational insight (*vide infra*).

Finally, it is interesting to compare the pre-edges of the V XAS spectra to those of other transition metals commonly found in biology. As an early first-row element, vanadium d-electron counts are generally lower for biologically relevant oxidation states, compared to iron or manganese [6]. Thus, an increased number of transitions into unoccupied 3*d* orbitals promotes pre-edge intensity. ${\mathrm{V_2O_5}}$ has a pre-edge intensity of 198 units (Table 2), while, e.g., the high-spin Fe(III) complex ${\mathrm{[Fe_2O(OAc)_2\{[OP(OEt)_2]_3Co(C2H5)\}_2]}}$ has a pre-edge intensity of 13.9 units; an intensity difference of roughly a factor of 14 [29]. Importantly, both metal sites are exclusively ligated by oxygenic ligands in an octahedral geometry, with relatively similar bond lengths [Fe–O average (range): 2.01 Å (1.79–2.14) [69], V–O: 1.96 Å (1.59–2.79); Table 1]. As discussed above, however, there are many factors that govern pre-edge intensity. Reducing the number of possible transitions as one moves across the periodic table is but one of these considerations.

In the octahedral limit, addition of electrons to the vanadium 3*d* manifold generally results in population of ${{t_{2g}}}$ orbitals, rather than the antibonding ${e_g}$ as in later first-row metals. This has a less significant impact on metal–ligand bond lengths; an important component of pre-edge intensity (*vide supra*). Additionally, while reduction from a ${\mathrm{d^{9}}}$ Cu(II) center to ${\mathrm{d^{10}}}$ Cu(I) occupies all the 3*d* orbitals, quenching pre-edge intensity, the low *d*-electron counts of vanadium ions provide a means for pre-edge intensity even in lower oxidation states. Again in the octahedral limit, reduction from V(IV) to V(III) quenches only one of the nine 1*s*$\rightarrow$ 3*d* excitations, or roughly 11 %. As a cautionary note, however, this picture breaks down in lower symmetry systems, where the particular d-orbital that is occupied (or vacated) in an oxidation state change can significantly alter the expected impact on the pre-edge intensity. Population of a 3*d* orbital with minimal *p*–*d* mixing should have a negligible impact on pre-edge intensity, while in contrast occupation of a 3*d* orbital with substantial *p*–*d* mixing should significantly decrease the pre-edge intensity. This distinction further emphasizes the need for careful interpretation of pre-edge intensities aided by computational methods, rather than reliance on simple, qualitative trends.

### XES spectra

As discussed above, the K$\beta$ XES spectrum arises from $n=3\rightarrow$1*s* fluorescence. The intense, dipole-allowed K$\beta$ mainline is due to electronic relaxation from the 3*p* shell, giving a ${{1s^{2}3p^{5}3d^{n}}}$ final state. However, the transition energy of the K$\beta$ mainline is not solely dependent on the 1*s* and 3*p* binding energies; instead, multiple additional factors impact the observed spectral features. Most significantly, the unpaired 3*p* electron can exchange couple to unpaired 3*d* electrons, giving rise to a series of pseudo-atomic multiplets of discrete energies. Charge-transfer states and 3*p* spin-orbit coupling (SOC) can additionally contribute to the features of the K$\beta$ mainline region [34, 70–72].

For first-row transition metals with high multiplicities, e.g., high-spin Fe(III) and Mn(II), the energetic splitting ($\Delta E_{\text {main}}$) of the K$\beta$ mainline features, the K$\beta _{1,3}$ and K$\beta '$, can be as large as 15 eV, due primarily to 3*p*–3*d* exchange coupling [34, 51, 70]. Furthermore, we have recently shown that the magnitude of $\Delta E_{\text {main}}$ can be significantly influenced by metal–ligand covalency, within a series of high-spin ferric complexes [34]. Increased covalency delocalizes the 3*d* spin density via the Nephelauxetic effect, which in turn decreases 3*p*–3*d* exchange and the mainline splitting [37]. For lower multiplicity metal ions such as those in the present work, 3*p*–3*d* exchange is significantly diminished.

**Fig. 3:**
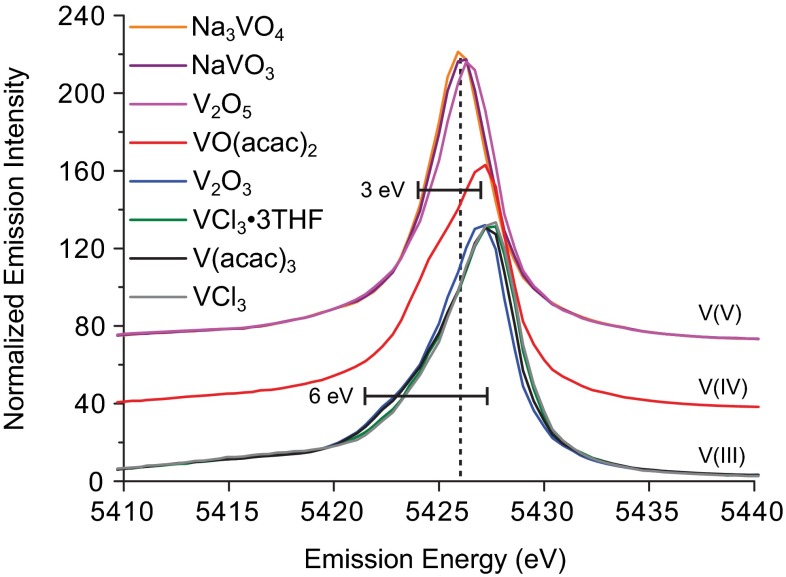
Mainline regions of the K$\beta$ XES spectra. The *dashed vertical line* is a visual guide for the shift in peak maxima to higher energy upon reduction. The *horizontal markers* show the expected magnitude of 3*p*–3*d* exchange, corresponding to 3 eV per unpaired electron, and the right edges are aligned with the average K$\beta _{1,3}$ maximum for the V(IV) and V(III) oxidation states

Figure 3 shows the K$\beta$ mainline regions of several complexes in this study.

As highlighted by the vertical dashed line, a decrease in vanadium oxidation state is found to result in K$\beta _{1,3}$ transitions shifted to higher energies. However, reduction of the vanadium ion concomitantly increases the 3*d* electron count, and thus the magnitude of the 3*d*–3*d* exchange coupling. Therefore, the observed shift in energy cannot conclusively be attributed to changing oxidation state. In fact, the K$\beta _{1,3}$ energy of vanadium metal is 5427.3 eV [73]; within the range of the V(III)–V(V) complexes reported herein (Table 3).

**Table 3:** Comparison of experimental and calculated XES parameters

Compound	Experimental	Experimental VtC		Calculated VtC	
	Mainline energy (eV)	Energy (eV)	Intensity ($\times 10^{-1}$)	Energy (eV)	Intensity
${\mathrm{Na_3VO_4}}$	5426.1	5447.8, 5464.4	69.4	5448.3, 5462.6	69.5
${\mathrm{NaVO_3}}$	5426.1	5447.3, 5463.8	71.1	5448.3, 5462.6	69.5
${\mathrm{V_2O_5}}$	5426.3	5448.1, 5462.9	58.5	5448.7, 5462.1	68.1
${\mathrm{VO(acac)_2}}$	5427.0	5446.8, 5458.0	51.8	5448.3, 5461.7	56.6
${\mathrm{V_2O_3}}$	5427.0	5441.5, 5458.5	45.7	5449.3, 5463.1	47.3
${\mathrm{VCl_3 * \, 3THF}}$	5427.4	5445.7, 5462.6	38.8	5451.4, 5462.0	38.9
${\mathrm{V(acac)_3}}$	5427.3	5446.0, 5456.8	47.2	5443.9, 5457.7	48.4
${\mathrm{VCl_3}}$	5427.4	5450.0, 5462.0	35.1	5451.1, 5462.0	36.0

Calculated IWAEs have been shifted 141.9 eV from the calibration in Fig. 6

Using a rough approximation of exchange energy corresponding to 3 eV per unpaired electron [34, 51], a coupling of around 3 and 6 eV is expected for the V(IV) and V(III) complexes, respectively. The horizontal markers in Fig. 3 show that subtle but distinct features corresponding to roughly the correct exchange stabilization energies are present in the spectra. Interestingly, taking the average energy of the 3*p*–3*d* exchange-split states (the center of the horizontal markers) to reflect the relative 3*p* energies in the absence of exchange coupling, a trend of higher transition energies for more *oxidized* vanadium ions is observed. This is consistent with a larger ${{Z}_{\text{eff}}}$ for the more oxidized ions, as we have previously observed in a series of vanadium L-edge (2p) XAS spectra [25].

It should be noted that despite the absence of exchange coupling in the 3${{d^{0}}}$ V(V) compounds in this study, differences among them, in both spectral shape and energy, are still observed. As mentioned above, these could be attributed to either charge-transfer states or to 3*p* SOC [32, 70]. In the latter case, the stabilizing effect is roughly an order of magnitude smaller than the 2*p* SOC, which can be determined from the vanadium K$\alpha$ emission line (2*p*$\rightarrow$ 1*s*, 7.6 eV [73]). Therefore, 3*p* SOC is conservatively placed at less than 1 eV. Orbital angular momentum is quenched upon orbital delocalization, and as evidenced by the experimental pre-edge (Table 2) and VtC intensities (Table 3), ${\mathrm{Na_3VO_4}}$ and ${\mathrm{NaVO_3}}$ have greater 3*p* and 4*p* mixing into both the vanadium 3*d* and the ligand valence orbitals compared to ${\mathrm{V_2O_5}}$. Additionally, compared to previous studies at later first-row metals such as iron [40], where both the metal 3*p* and 4*p* orbitals contribute to VtC intensity via mixing with the ligand orbitals, the 3*p* orbitals of vanadium should play an increased role. Therefore, the less localized 3*p* character of ${\mathrm{Na_3VO_4}}$ and ${\mathrm{NaVO_3}}$ results in decreased orbital angular momentum and a smaller SOC contribution. A loss of spin-orbit stabilization should lower the energy of the mainline transitions, consistent with the spectra shown in Fig. 3.

To higher energy of the mainline, the VtC region can provide detailed insight into the occupied ligand valence orbitals, often from a simple molecular orbital picture [41]. As discussed previously, VtC peaks can be understood as a valence to metal 1*s* transition, and thus the transition energies provide a direct probe of the relative binding energies of the valence orbitals [42]. Similar to the pre-edge region of the XAS spectrum, the intensity mechanism in the VtC region is almost exclusively electric dipole and, therefore, the amount of metal *p* character mixed into the donor ligand orbital has been found to strongly correlate with VtC intensity [38–40].

**Fig. 4:**
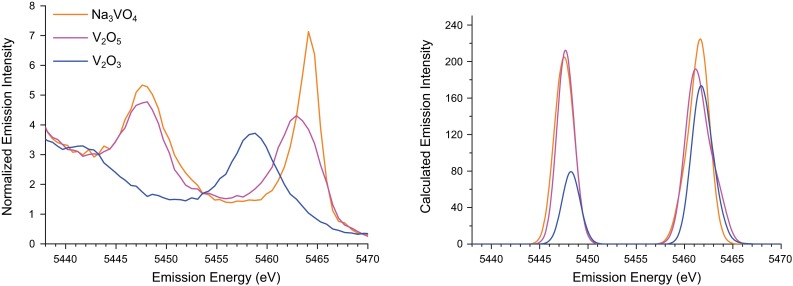
Experimental (*left*) and calculated (*right*) VtC regions of the K$\beta$ XES spectra of ${\mathrm{Na_3VO_4}}$, ${\mathrm{V_2O_5}}$, and ${\mathrm{V_2O_3}}$. Calculated VtC spectra are shifted by 141.9 eV

The K$\beta$ VtC XES spectra of ${\mathrm{V_2O_3}}$, ${\mathrm{V_2O_5}}$, and ${\mathrm{Na_3VO_4}}$ are shown in Fig. 4. The highest energy K$\beta _{2,5}$ features, which are due to ligand *p*-type orbitals [41], must arise from oxygen 2*p* orbitals. The sharp peak in the ${\mathrm{Na_3VO_4}}$ spectrum indicates more homogeneous transition energies than in the case of ${\mathrm{V_2O_5}}$. Additionally, the spectral features of ${\mathrm{V_2O_5}}$ are shifted to slightly lower energies. Perturbations to the oxide 2*p* energies are expected to occur upon changes in bonding, in particular due to bond lengths. Decreased Coulombic attraction of the oxide 2*p* electrons to the ${\mathrm{V^{5+}}}$ ion in ${\mathrm{V_2O_5}}$, due to a longer bond length, should destabilize the oxygen 2*p* orbitals, resulting in transitions shifted to lower energy. Thus, the longer average V–O bonds in ${\mathrm{V_2O_5}}$, by 0.37 Å (Table 1), cause the VtC transitions to shift to lower energies. Furthermore, the deviation in V–O bond length in ${\mathrm{V_2O_5}}$ (1.2 Å) is significantly larger than that of ${\mathrm{Na_3VO_4}}$ (0.04 Å). This heterogeneity in bond lengths results in a larger distribution of transition energies, which is manifest in the broader shape of the K$\beta _{2,5}$ peak.

Compared to ${\mathrm{V_2O_5}}$ and ${\mathrm{Na_3VO_4}}$, the K$\beta ''$, which is due to O 2*s* orbitals, and K$\beta _{2,5}$ peaks of the vanadium(III) oxide ${\mathrm{V_2O_3}}$ are shifted to substantially lower energy, by approximately 6 eV. As discussed above, the longer average bond length (by 0.05 Å) constitutes a good rationale for this observation. Additionally, a two-electron reduction lowers the vanadium effective nuclear charge ($Z_{\text {eff}}$) experienced by the oxygenic ligands. This further serves to raise the orbital energies, and thus decrease the transition energy. This is consistent with previous studies that found a one-electron reduction of iron would shift the VtC peaks of structurally analogous complexes to lower energy by approximately 1.2 eV [40]. However, in the case of manganese, a similar reduction resulted in peak energies that were shifted to *higher* energy by about 0.2 eV [38].

There are additionally clear trends observed in the intensities of the VtC features. While the K$\beta _{2,5}$ peak height of ${\mathrm{Na_3VO_4}}$ is substantially larger than ${\mathrm{V_2O_5}}$, the increased width of the latter feature results in comparable integrated intensities between the two (Table 3). The tetrahedral geometry of ${\mathrm{Na_3VO_4}}$ results in shorter V–O bonds, however, which should increase VtC intensity [40, 41]. While the individual O$\rightarrow$V 1*s* transition moments are larger for ${\mathrm{Na_3VO_4}}$, the presence of two additional ligands (and the accompanying transitions) in ${\mathrm{V_2O_5}}$ seemingly offsets the shorter bond lengths. The K$\beta ''$ feature shows a definite sensitivity to V–O bond length however, as the peak intensity in the ${\mathrm{V_2O_5}}$ spectrum is lower despite the two additional ligands, and the K$\beta ''$ of ${\mathrm{V_2O_3}}$ is much weaker. Given the contracted nature of the oxygen 2s atomic orbitals, changes in bond length should have a larger impact on metal *p* mixing into the 2*s*, compared to the more diffuse 2*p*.

**Fig. 5:**
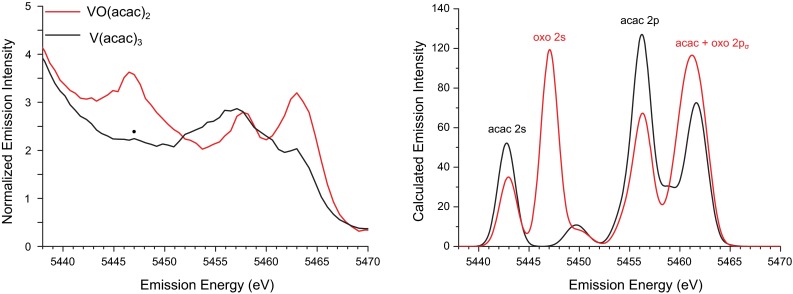
Experimental (*left*) and calculated (*right*) VtC regions of the K$\beta$ XES spectra of ${\mathrm{VO(acac)_2}}$ and ${\mathrm{V(acac)_3}}$. The $\cdot$ denotes the K$\beta ''$ feature of ${\mathrm{V(acac)_3}}$ determined from the first spectral moment. Calculated VtC spectra are shifted by 141.9 eV, and the major orbital contributions to the calculated transitions are assigned

Whereas EXAFS is sensitive to the average radial distributions of scatters with similar *Z*, VtC XES can differentially probe specific metal–ligand bonds, even with the same ligating atom. Comparison of the VtC XES spectra of ${\mathrm{VO(acac)_2}}$ and ${\mathrm{V(acac)_3}}$ in Fig. 5 reveals higher energy K$\beta _{2,5}$ regions of similar intensity, whereas the K$\beta ''$ peak at about 5447 eV in the spectrum of ${\mathrm{VO(acac)_2}}$ is clearly absent in the ${\mathrm{V(acac)_3}}$ spectrum. Unlike the XAS pre-edge region, low centrosymmetry is not required for VtC intensity, because in an octahedral geometry, ligand orbitals which interact with the metal in a $\sigma$ fashion can mix with the metal *p* [39]. The ${\mathrm{acac^{-}}}$ valence orbitals, which are a combination of oxygen and carbon 2*p* orbitals, therefore give rise to K$\beta _{2,5}$ regions of comparable intensity in both spectra.

The K$\beta ''$ peak is due to ligand orbitals that are dominantly *s*-type, thus this feature should correspond to an O 2*s* atomic orbital. Close examination reveals that the ${\mathrm{V(acac)_3}}$ spectrum also has a K$\beta ''$ feature at similar energy. As both metals are ligated entirely by oxygen atoms, VtC transitions from O 2*s* orbitals are in fact expected in both spectra. The vastly increased intensity in the case of the ${\mathrm{VO(acac)_2}}$ is due to the markedly different bonding of the vanadyl oxo compared to the acac ligand. The short, covalent oxo bond results in considerably more vanadium *p* mixing with the oxo 2*s*, and increased transition intensity. Similarly, the increased intensity of the highest energy K$\beta _{2,5}$ feature in the ${\mathrm{VO(acac)_2}}$ indicates stronger bonding with orbitals that are O 2*p* in character. Considering that ligand orbitals require a $\sigma$ orientation to interact with metal *p* orbitals, this peak can likely be attributed to the vanadyl O ${{2p_\sigma }}$ orbital.

### Spectral calculations

#### VtC XES

To obtain additional insight into the trends observed in these data, the VtC XES spectra were calculated using a well-established ground-state DFT method [38–40, 42]. The calculated spectra corresponding to the examples discussed previously are shown in Figs. 4 and 5, and satisfactory reproduction of the experimental trends are found. Interestingly, the calculations fail to capture the shift to lower energy of the ${\mathrm{V_2O_3}}$ features compared to ${\mathrm{V_2O_5}}$. A similar shortcoming was found in our previous manganese study [38]. This suggests that there may be contributions to the observed oxidation state trends, e.g., core-hole relaxation effects, which are not captured within this computational model. Examination of the calculated ${\mathrm{V(acac)_3}}$ and ${\mathrm{VO(acac)_2}}$ spectra (Fig. 5) reveals good agreement between calculated and experimental energies and intensities. Consistent with the interpretation of the experimental data, the large K$\beta ''$ peak in the ${\mathrm{VO(acac)_2}}$ spectrum arises from a transition from the vanadyl oxo 2s atomic orbital to the vanadium 1*s*. The short, covalent nature of this bond increases vanadium *p* mixing into the oxo 2*s* and thus the transition intensity.

**Fig. 6:**
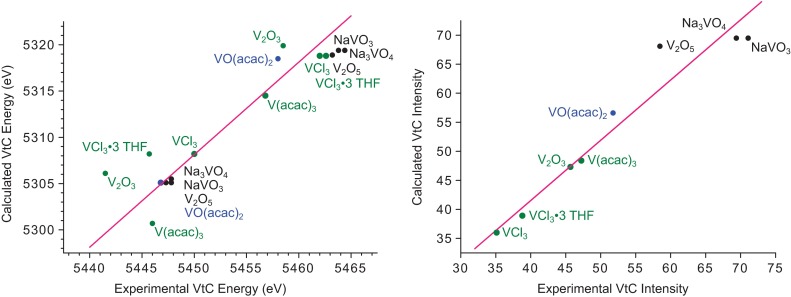
Correlations between experimental and calculated VtC energies (*left*) and intensities (*right*). Markers are *color coded* by vanadium oxidation state, with *green* ${\mathrm{V^{3+}}}$, *blue* ${\mathrm{V^{4+}}}$, and *black* ${\mathrm{V^{5+}}}$. Equations for the linear fits: $E_{\mathrm{calc}} = E_{\mathrm{exp}} - 141.9\ (R^2 _{\mathrm{adj}} = 0.84)$, $I_{\mathrm{calc}} = 1.04\times I_{\mathrm{exp}}\ (R^2 _{\mathrm{adj}} = 0.99)$

As with previous work in the case of iron [40], manganese, [38] and chromium, [45] the accuracy of the selected computational method can be evaluated by quantitative comparison of the experimental and calculated transition energies and intensities. The linear correlations shown in Fig. 6 demonstrate in particular the high fidelity of the calculated transition intensities, derived from the calculated vanadium *p*-orbital character found in the donor ligand orbitals. The larger VtC intensity upon increasing oxidation state is rationalized by the generally shorter metal–ligand bonds, which promotes vanadium *p*-orbital mixing with filled ligand orbitals. Additionally, the intercept and slope of the energy and intensity correlations, respectively, provide correction factors used for this computational protocol (*vide supra*).

#### XAS pre-edges

The nature of the pre-edge transitions observed herein was also investigated using TD-DFT-calculated XAS spectra. As shown in Fig. 1, experimental differences in intensity and energy between ${\mathrm{V_2O_3}}$, ${\mathrm{V_2O_5}}$, and ${\mathrm{Na_3VO_4}}$ are well reproduced. The largest intensity of the ${\mathrm{Na_3VO_4}}$ pre-edge, due to the tetrahedral geometry, is correctly calculated, as is the lower energy pre-edge and diminished intensity of ${\mathrm{V_2O_3}}$ given the lower oxidation state and longer bond lengths. Figure 2 shows the calculated pre-edge spectra of ${\mathrm{V(acac)_3}}$ and ${\mathrm{VO(acac)_2}}$, which are also in very good agreement with the experimental data. As discussed previously, the octahedral ${\mathrm{V(acac)_3}}$ has minimal transition dipole intensity, as mechanisms for 4*p* mixing are effectively quenched, while the square pyramidal geometry and highly covalent vanadium-oxo bond provide ample 4*p* mixing in ${\mathrm{VO(acac)_2}}$. The 1*s*$\rightarrow$3*d* pre-edge excitations can directly occur via the quadrupole transition operator, however, though these are roughly two orders of magnitude less intense than a fully allowed electric dipole transition [26, 29]. Examination of the calculated pre-edge spectrum of ${\mathrm{V(acac)_3}}$ reveals that roughly half of the spectral intensity derives from quadrupole transitions, and furthermore that the featured, albeit weak, experimental pre-edge is reproduced with high fidelity. Thus, even without a mechanism for *p*–*d* mixing and significant pre-edge intensity, meaningful insight can still be extracted from the pre-edge region using the TD-DFT method.

**Fig. 7:**
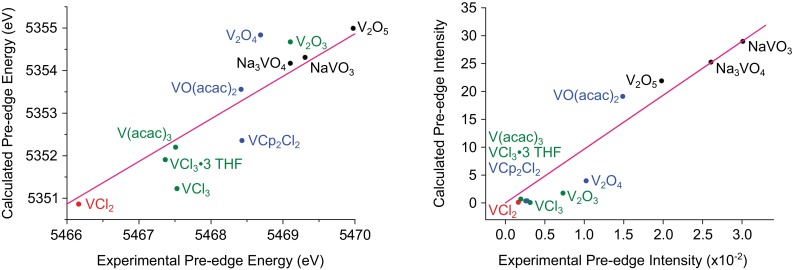
Correlations between experimental and calculated XAS pre-edge energies (*left*) and intensities (*right*). Markers are *color coded* by vanadium oxidation state, with *red* ${\mathrm{V^{2+}}}$, *green* ${\mathrm{V^{3+}}}$, *blue* ${\mathrm{V^{4+}}}$, and *black* ${\mathrm{V^{5+}}}$. Equations for the linear fits: $E_{\mathrm{calc}} = E_{\mathrm{exp}} - 115.1\ (R^2 _{\mathrm{adj}} = 0.80)$, $I_{\mathrm{calc}} = (9.7\times 10^{-2}) I_{\mathrm{exp}}\ (R^2 _{\mathrm{adj}} = 0.95)$

Similar to the XES calculations above, comparison of the experimental and calculated energies and intensities reveals good agreement (Fig. 7), providing evidence for the success of the TD-DFT method.

Thus, the insight obtained from the calculations above can be taken to represent the experimental findings with reasonable confidence. In particular, the calculations illustrate that XAS pre-edge intensity should be interpreted with regard to metal *p*–*d* mixing, rather than utilizing geometry and oxidation state. For example, ${\mathrm{VCp_2Cl_2}}$ and ${\mathrm{VO(acac)_2}}$ both have ${\mathrm{V^{4+}}}$ ions. The former has a four-coordinate, distorted tetrahedral geometry, while ${\mathrm{VO(acac)_2}}$ is a five-coordinate, distorted square pyramid. A conventional interpretation would likely lead to the conclusion that ${\mathrm{VCp_2Cl_2}}$, being lower coordinate and with less centrosymmetry, should have considerably higher pre-edge intensity. In fact, the opposite is observed in the experimental data, and the TD-DFT calculations are in excellent agreement. While elucidating the exact cause of this observation is challenging, the highly covalent nature of the vanadyl unit compared to the ligand bonds in ${\mathrm{VCp_2Cl_2}}$ is a likely contributor.

## Conclusions

This study illustrates the complementary nature of vanadium XAS and XES, and provides archetypal methods for interpretation of the spectroscopic data using both fundamental principles and theoretical calculations. The sensitivity of VtC XES to the nature of metal–ligand bonding, and not solely the identity of the ligand or its bond length, renders it a powerful tool for the study of fundamental tenets of coordination chemistry. Differentiating molecular orbitals arising from two “types” of the same ligating atom, as demonstrated herein, is a significant advantage considering this method is easily applicable to metalloenzyme systems. The XAS data demonstrate multiple ways in which the extent of metal* p*–*d* mixing is modulated, including geometry, covalency, and oxidation state. Consideration of all factors, ideally with the aid of DFT calculations, is key for correctly interpreting spectral properties such as pre-edge intensity. A combined XAS and XES study allows interrogation of both occupied and virtual metal orbitals, as well as ligand-based MOs which XAS typically cannot probe. Such methods, broad in their applicability and robust in their interpretation, are powerful tools for the continued investigation of transition metal catalyzed reactions in complex, biological systems.

## Dedication

This work is dedicated to Professor Edward I. Solomon on the occasion of his receipt of the 2016 Alfred Bader Award.

## Electronic supplementary material

Below is the link to the electronic supplementary material.

Supplementary material 1 (PDF 389 kb)
