# Supplementary material for: Experimental and theoretical correlations between vanadium K-edge X-ray absorption and Kβ emission spectra
Source: J Biol Inorg Chem. 2016 Jun 1;21:793–805. doi: 10.1007/s00775-016-1358-7 (PMC4989026; doi:10.1007/s00775-016-1358-7)
Supplement: Supplementary file 1 — Supplementary material 1 (PDF 389 kb) [file 775_2016_1358_MOESM1_ESM.pdf]

Supplementary Information

Experimental and Theoretical Correlations Between Vanadium K-edge X-Ray Absorption and Kβ Emission Spectra

Julian A. Rees[§a,b], Aleksandra Wandzilak[§a,c], Dimitrios Maganas[a], Nicole I. C. Wurster[a], Stefan Hugenbruch[a], Joanna K. Kowalska[a], Christopher J. Pollock[‡a], Frederico A. Lima[e], Kenneth D. Finkelstein[f] and Serena DeBeer (🖂)[a,d]

[a]*Max-Planck-Institut für Chemische Energiekonversion, Stiftstr. 34-36, 45470 Mülheim an der Ruhr, Germany*
[b]*Department of Chemistry, University of Washington, Box 351700, Seattle, WA 98195-1700, United States*
[c]*AGH University of Science and Technology, Faculty of Physics and Applied Computer Science, al. Mickiewicza 30, 30-059 Kraków, Poland*
[d]*Department of Chemistry and Chemical Biology, Cornell University, Ithaca, NY 14853, United States*
[e]*Centro Nacional de Pesquisa em Energia e Materiais, , Laboratório Nacional de Luz Síncrotron, Rua Giuseppe Máximo Scolfaro, 10000 13083-970, Campinas, SP, Brazil*
[f]*Cornell High Energy Synchrotron Source, Wilson Laboratory, Cornell University, Ithaca, NY 14853, United States*

In honor of Professor Edward I. Solomon's 2016 ACS Alfred Bader Award

[§]These authors contributed equally to this work
[‡]Current address: Department of Chemistry, The Pennsylvania State University, University Park, PA 16802, United States
🖂 serena.debeer@cec.mpg.de

List of Figures

S1 Overlay of experimental XAS spectra . . . 3
S2 Experimental XAS spectra and fits of the V(V) compounds . . . 3
S3 Experimental XAS spectra and fits of the V(IV) compounds . . . 4
S4 Experimental XAS spectra and fits of the V(III) compounds . . . 4
S5 Experimental XAS spectrum and fit of VCl_2 . . . 5
S6 Experimental XES spectra and fits of Na_3VO_4 and $NaVO_3$ . . . 6
S7 Experimental XES spectra and fits of V_2O_5 and V_2O_3 . . . 6
S8 Experimental XES spectra and fits of $V(acac)_3$ and $VO(acac)_2$ . . . 7
S9 Experimental XES spectra and fits of VCl_3 and $VCl_3 \cdot 3\,THF$ . . . 7

1 XAS Data

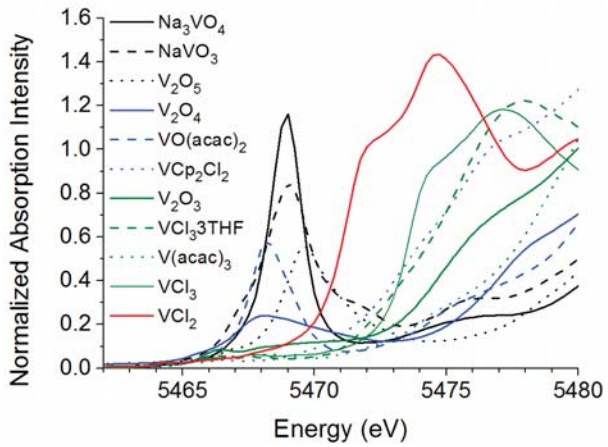

Fig. S1 Overlay of experimental XAS spectra showing the full edge region and colored by oxidation state

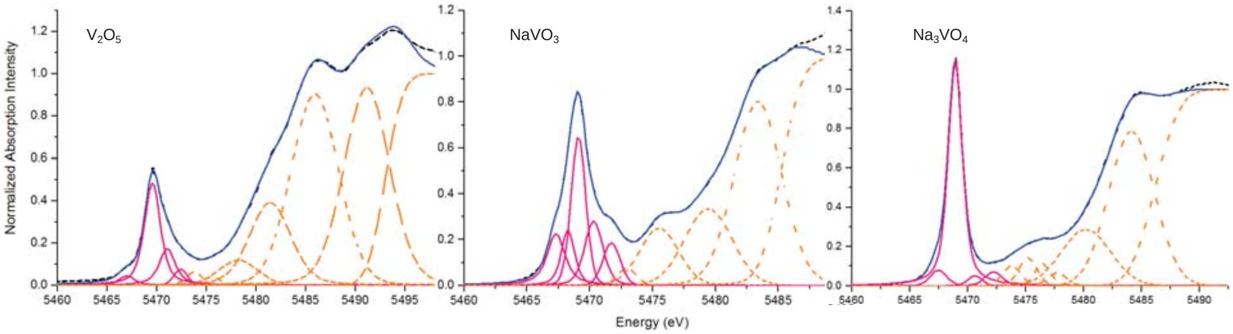

Fig. S2 Experimental XAS spectra and fits of the V(V) compounds. Black dashes are experimental data, blue trace is total fit, and magenta peaks are those included in the pre-edge area

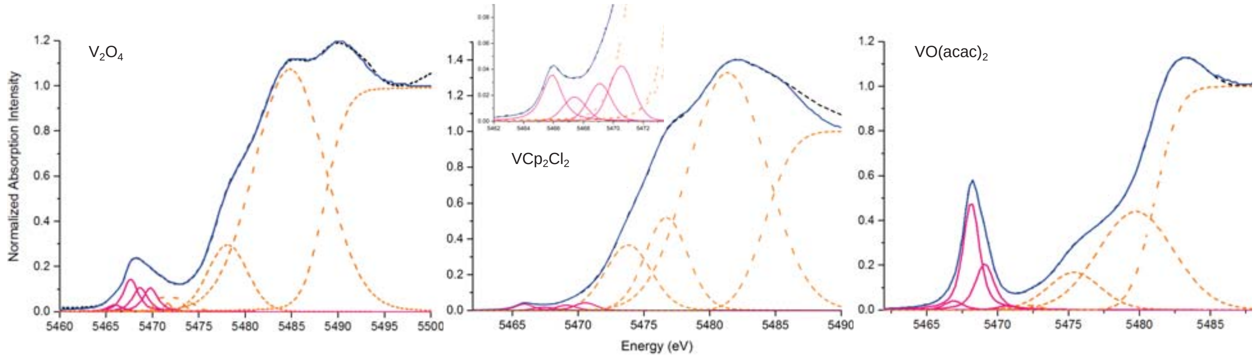

Fig. S3 Experimental XAS spectra and fits of the V(IV) compounds. Black dashes are experimental data, blue trace is total fit, and magenta peaks are those included in the pre-edge area

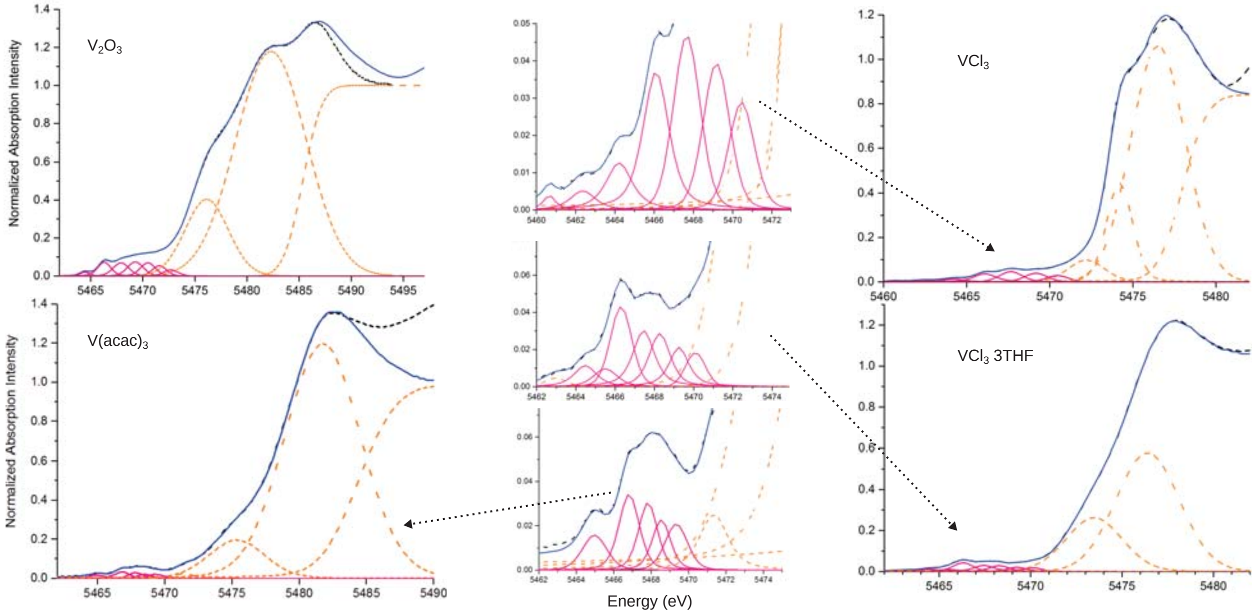

Fig. S4 Experimental XAS spectra and fits of the V(III) compounds. Black dashes are experimental data, blue trace is total fit, and magenta peaks are those included in the pre-edge area. The central column shows enlargements of the pre-edge regions for selected compounds

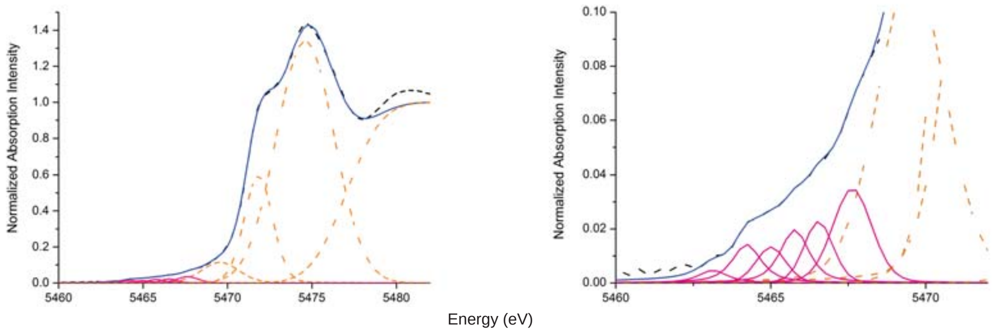

Fig. S5 Experimental XAS spectrum and fit of VCl_2. Black dashes are experimental data, blue trace is total fit, and magenta peaks are those included in the pre-edge area. Left: complete XANES region. Right: enlargement of the pre-edge region

2 XES Data

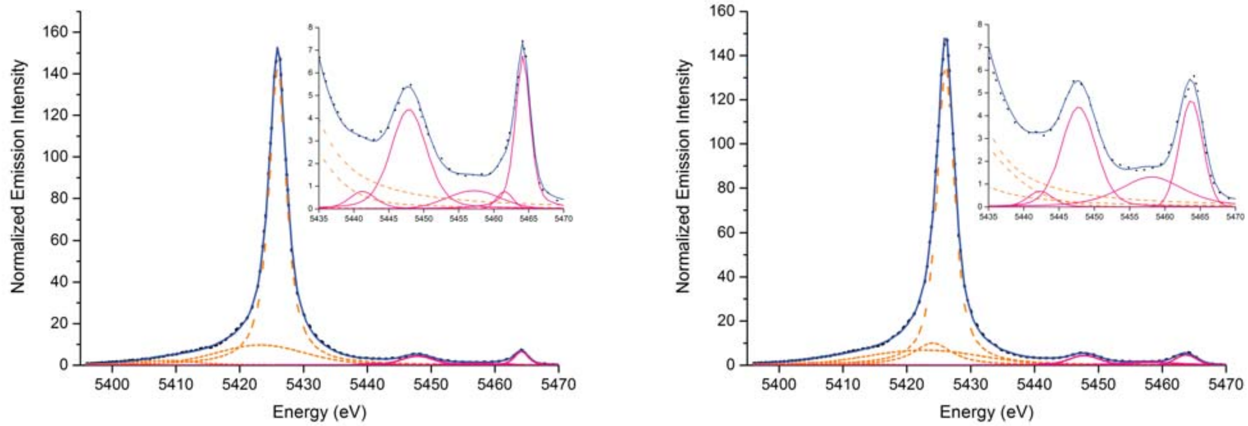

Fig. S6 Experimental XES spectra and fits of Na_3VO_4 (left) and $NaVO_3$ (right). Black dashes are experimental data, blue trace is total fit, and magenta peaks are those included in the VtC area. Inset: enlargment of the VtC region

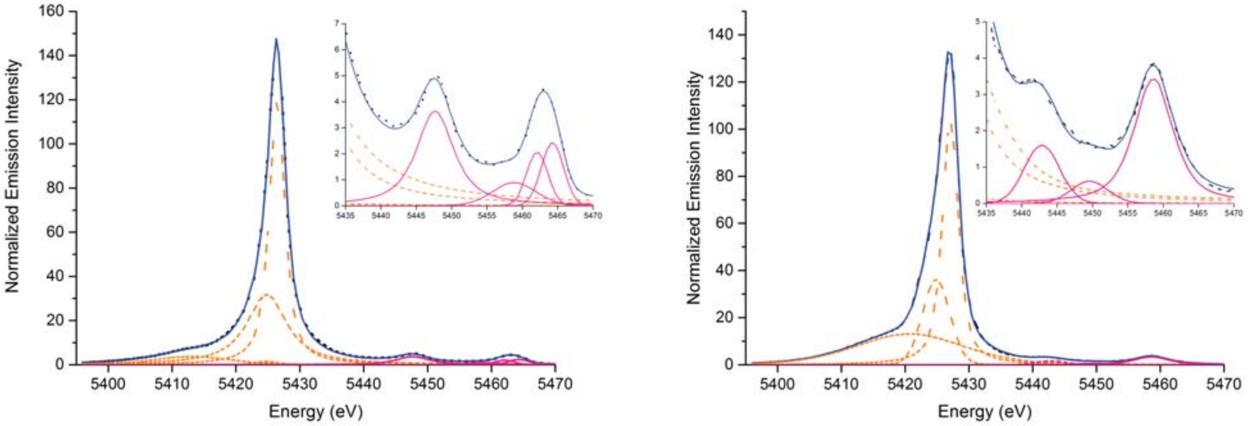

Fig. S7 Experimental XES spectra and fits of V_2O_5 (left) and V_2O_3 (right). Black dashes are experimental data, blue trace is total fit, and magenta peaks are those included in the VtC area. Inset: enlargment of the VtC region

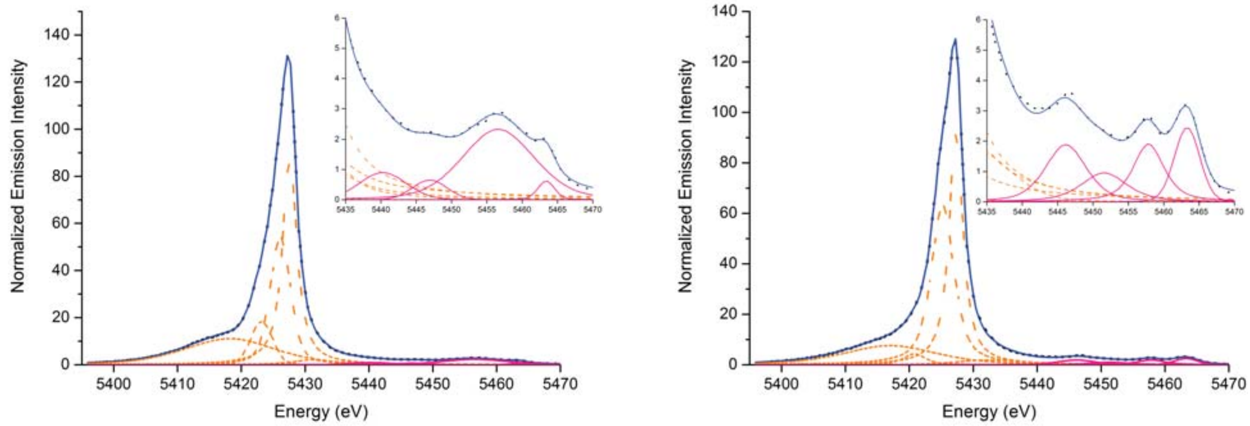

Fig. S8 Experimental XES spectra and fits of $V(acac)_3$ (left) and $VO(acac)_2$ (right). Black dashes are experimental data, blue trace is total fit, and magenta peaks are those included in the VtC area. Inset: enlargment of the VtC region

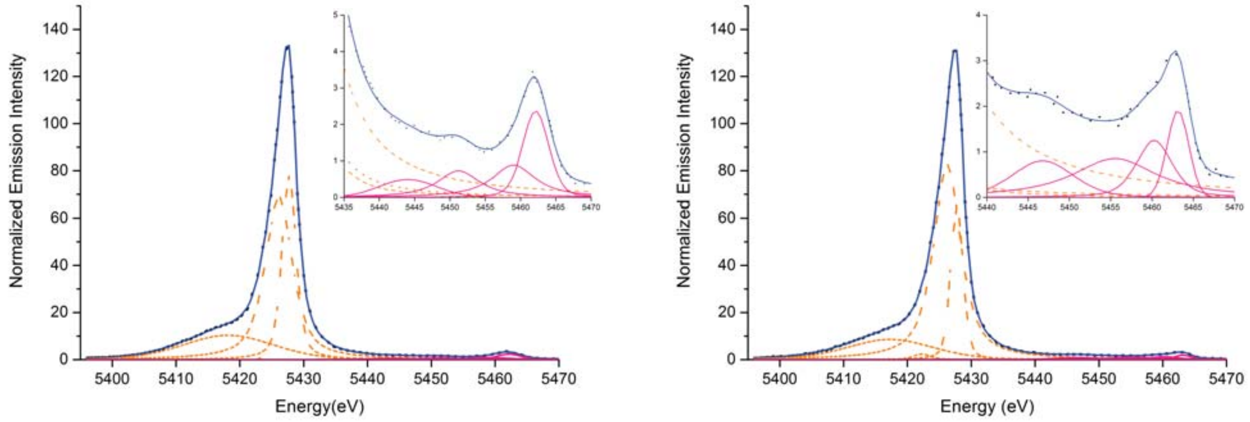

Fig. S9 Experimental XES spectra and fits of VCl_3 (left) and $VCl_3 \cdot 3\,THF$ (right). Black dashes are experimental data, blue trace is total fit, and magenta peaks are those included in the VtC area. Inset: enlargment of the VtC region

3 Sample Input Files

The following are representative input files for the geometry optimizations and spectral calculations of molecular and extended lattice compounds.

A generic geometry optimization was performed using the following input:

```
! UKS Opt BP86 def2-TZVP def2-TZV/J TightSCF
! VerySlowConv Normalprint PAL8

%maxcore 4000

* xyzfile 0 3 V_acac3.xyz
```

An XES calculation was performed using the following input:

```
! UKS BP86 def2-TZVP def2-TZV/J TightSCF
! VerySlowConv Normalprint PAL8

%maxcore 4000

%method
 SpecialGridAtoms 23
       SpecialGridIntAcc 7
end

%xes
  CoreOrb 0,0
 OrbOp 0,1
end

* xyzfile 0 3 V_acac3.xyz
```

An XAS calculation, in this case showing the protocol for an extended lattice solid, was performed using the following input:

```
! UKS B3LYP def2-TZVP def2-TZV/J TightSCF
! VerySlowConv Normalprint PAL8

%pointcharges "V2O3.pc"

%scf
```

```
  MaxIter=2000
end

%maxcore 4000

%method
  SpecialGridAtoms 23
        SpecialGridIntAcc 7
end

%tddft
   NRoots 50
   MaxDim 150
  OrbWin[0]=0,0,-1,-1
   OrbWin[1]=0,0,-1,-1
   DoQuad true
end

* xyz -14 5
O  0.917 10.01 15.179
O  3.257 8.66 15.179
O  5.871 10.01 15.179
O  3.257 11.361 15.179
O  5.733 12.95 15.179
O  1.697 8.501 12.844
O  4.174 10.09 12.844
O  4.174 12.791 12.844
O  1.559 11.44 12.844
O  6.513 11.44 12.844
V  2.477 10.01 13.824
V  4.954 11.44 14.198
V> 0.7496 0 8.58 16.159 NewECP "SD(10,MDF)" end
V> 0.7496 2.477 10.01 16.534 NewECP "SD(10,MDF)" end
V> 0.7496 4.954 8.58 16.159 NewECP "SD(10,MDF)" end
V> 0.7496 7.431 10.01 16.534 NewECP "SD(10,MDF)" end
V> 0.7496 2.477 12.87 16.159 NewECP "SD(10,MDF)" end
V> 0.7496 4.954 14.3 16.534 NewECP "SD(10,MDF)" end
V> 0.7496 7.431 12.87 16.159 NewECP "SD(10,MDF)" end
V> 0.7496 2.477 7.15 11.489 NewECP "SD(10,MDF)" end
V> 0.7496 0 8.58 11.863 NewECP "SD(10,MDF)" end
V> 0.7496 0 11.44 11.489 NewECP "SD(10,MDF)" end
V> 0.7496 4.954 8.58 11.863 NewECP "SD(10,MDF)" end
V> 0.7496 4.954 11.44 11.489 NewECP "SD(10,MDF)" end
V> 0.7496 2.477 12.87 11.863 NewECP "SD(10,MDF)" end
V> 0.7496 7.431 12.87 11.863 NewECP "SD(10,MDF)" end
V> 0.7496 2.477 7.15 14.198 NewECP "SD(10,MDF)" end
V> 0.7496 0 11.44 14.198 NewECP "SD(10,MDF)" end
V> 0.7496 7.431 10.01 13.824 NewECP "SD(10,MDF)" end
V> 0.7496 4.954 14.3 13.824 NewECP "SD(10,MDF)" end
*
```
